# Repeatability and heritability of inhibitory control performance in wild toutouwai (*Petroica longipes*)

**DOI:** 10.1098/rsos.231476

**Published:** 2023-11-15

**Authors:** Ella McCallum, Rachael C. Shaw

**Affiliations:** School of Biological Sciences, Te Herenga Waka Victoria University of Wellington, Wellington, New Zealand

**Keywords:** inhibitory control, repeatability, heritability, convergent validity, cognition, individual differences

## Abstract

Despite increasing interest in the evolution of inhibitory control, few studies have examined the validity of widespread testing paradigms, the long-term repeatability and the heritability of this cognitive ability in the wild. We investigated these aspects in the inhibitory control performance of wild toutouwai (North Island robin; *Petroica longipes*), using detour and reversal learning tasks. We assessed convergent validity by testing whether individual performance correlated across detour and reversal learning tasks. We then further evaluated task validity by examining whether individual performance was confounded by non-cognitive factors. We tested a subset of subjects twice in each task to estimate the repeatability of performance across a 1-year period. Finally, we used a population pedigree to estimate the heritability of task performance. Individual performance was unrelated across detour and reversal learning tasks, indicating that these measured different cognitive abilities. Task performance was not influenced by body condition, boldness or prior experience, and showed moderate between-year repeatability. Yet despite this individual consistency, we found no evidence that task performance was heritable. Our findings suggest that detour and reversal learning tasks measure consistent individual differences in distinct forms of inhibitory control in toutouwai, but this variation may be environmentally determined rather than genetic.

## 1. Introduction

Individuals often exhibit striking differences in their ability to suppress prepotent responses, referred to as inhibitory control [1–6]. Deficits in this executive function are associated with impulsive acts and reliance on outdated information or irrelevant stimuli, which can prevent an individual from reaching their goals [7,8]. In humans, superior inhibition is linked to better health, social and financial outcomes [9]. Likewise, this ability is thought to facilitate many fitness-relevant behaviours in non-human animals. For example, researchers have speculated that inhibition may allow animals to resist launching a premature attack on prey [10], cache food for later [11,12] or share resources with conspecifics [13]. The last decade has seen an upsurge of interest in the causes and consequences of individual variation in this ability [1,13–16]. However, to understand whether and how selection acts on inhibitory control, it is crucial to assess whether tasks used to assess inhibitory control are valid measures of repeatable, heritable individual differences in this ability in the wild [17,18].

In non-human animals, two of the most frequently assessed forms of inhibitory control are motor and cognitive inhibition. Motor inhibition describes the ability to withhold prepotent motor actions [8,19]. It is often measured using detour tasks, where individuals must inhibit reaching directly for a reward through a transparent barrier and instead detour to an opening [20]. By contrast, cognitive inhibition involves the suppression of unwanted thoughts and memories, including ‘intentionally forgetting’ information that is no longer accurate [7,21]. This can be assessed with reversal learning tasks, where animals learn to respond differently to different stimuli, then must inhibit acting on this association and learn a new one when reward contingencies are reversed [22]. Reversal learning tasks also measure cognitive flexibility, an executive function that allows animals to adjust to changing task demands and priorities [22,23]. Inhibitory control is a core component of cognitive flexibility, as modifying thoughts and behaviour requires the inhibition of a previous perspective or response [7]. As detour and reversal learning tasks require little equipment and can be adapted to a range of species, they are widely used in captivity and the field [1,3–5]. However, there are concerns that these tasks may not provide accurate assays of inhibitory control [3,4].

In a well-designed cognitive test battery, an individual's performances should be highly correlated across tasks measuring the same cognitive construct, a concept known as convergent validity [24]. Assessing convergent validity can indicate whether tasks measure the expected cognitive abilities and provide deeper insight into the structure of cognition [25]. Studies of non-human animals have often neglected convergent validity by using a single task or focusing on group-level performance rather than individual differences [25]. Where it has been assessed, individual performances have often shown no correlation across putative inhibitory control tasks [2,25–29], suggesting that these measure distinct abilities which are not underpinned by a ‘general’ inhibitory control ability. Understanding the structure of inhibitory control is vital to understand its evolution. If inhibitory control is best understood as a collection of independent abilities with different roles in guiding behaviour, then examining the fitness correlates of performance in a single task may result in misleading conclusions.

A lack of convergent validity can also arise if tasks measure unwanted variables [25]. Behavioural tasks can be confounded by non-cognitive factors which vary across species and task type. For example, pheasants (*Phasianus colchicus*) performed better on a detour task when they had experience with transparent objects, but body condition did not affect performance [3]. By contrast, toutouwai (*Petroica longipes*) in better condition—but not those with experience—performed better on detour task [4]. Personality, defined as individual behavioural differences that are consistent across time and context [30], can also influence cognitive task performance in unpredictable ways [31]. Higher levels of boldness have been linked to better detour performance [32,33] but worse reversal learning ability [34]. Bolder, neophilic and/or exploratory individuals may also be more likely to participate in voluntary tests or be less fearful and consequently perform better [35,36]. Investigating which factors contribute to task performance can improve the validity of measures [17,37]. Possible confounds can then be accounted for within tasks (e.g. by habituating animals to the apparatus) or by measuring variables in separate assays and controlling for them statistically [17].

Another crucial, but often neglected, step in validating cognitive tasks is assessing whether they measure consistent individual differences in performance [25,38]. Repeatability describes the proportion of phenotypic variation due to between-subject variation [39]. Cognitive repeatability has typically been measured over short timescales in captivity, which can bias results due to similarity in the genetic and environmental influences on performance [38,40]. Only a small number of studies have taken repeated measures of inhibitory control performance over long timescales in the wild. Detour task performance showed significant (albeit low) long-term repeatability in great tits (*Parus major*) [6], but not in toutouwai (*Petroica longipes*) [4]. Australian magpies (*Gymnorhina tibicen*) showed moderate long-term repeatability in a reversal learning task, but no significant repeatability in a detour task [41] (despite high short-term repeatability in both tasks [5]). Understanding the long-term consistency of inhibitory control in the wild is crucial, as the ability to interpret links between cognition and other traits such as personality, behaviour and fitness depends on whether an individual's cognition is temporally stable [42]. Moreover, repeatability can indicate the upper limit of heritability [39,43], a measure that describes the proportion of trait variation that is driven by genetic differences [44].

Exploring the genetic basis of cognitive variation in natural populations is crucial, as selection can only operate on heritable traits, and the speed of evolution partly depends on the strength of this heritability [43]. However, heritability estimates can be logistically difficult to obtain from natural populations [43]. While repeatability is a valuable substitute for heritability, it does not separate genetic and environmental contributions to individual variation [39,43]. A more reliable way to assess a trait's evolutionary potential is to measure narrow-sense heritability by testing related individuals and calculating the proportion of additive genetic variation [39]. The heritability of inhibitory control is well-studied in humans [45], showing a moderate to strong genetic component [45–48], but has received less attention in non-human animals, with estimates ranging from low and non-significant [49,50] to moderate [51–53]. To date, the heritability of cognitive traits has largely been assessed using laboratory populations of model species [18,54] which may not represent the genetic variation of their wild counterparts [55–58].

In this study, we assessed the validity, repeatability and heritability of inhibitory control task performance in wild toutouwai (North Island robin; *Petroica longipes*), a small, insectivorous passerine endemic to New Zealand. Both sexes cache food and males share food with their mates during the breeding season [59]. As both behaviours likely require individuals to overcome a prepotent impulse to eat food immediately, inhibitory control should be ecologically relevant to this species. We used detour reaching and reversal learning tasks to quantify individual differences in inhibitory control. We examined convergent validity by exploring how individual performances correlated across task type. To further assess task validity, we investigated whether boldness, body condition or prior experience confounded performance. We tested a subset of birds twice in each task to determine whether performance was repeatable across years. Finally, we measured the heritability of task performance using a population pedigree.

## 2. Methods

We conducted this study at Zealandia Ecosanctuary, a 225 ha fenced sanctuary in Wellington, New Zealand (41°18′ S, 174°44′ E). Subjects were colour-banded birds belonging to our long-term research population [60] and ranged from six months to 12 years of age. Toutouwai in Zealandia Ecosanctuary are protected from non-native mammalian predators by a predator exclusion fence but are exposed to native avian predators and natural weather conditions. Members of our research population are given small numbers of mealworms (*Tenebrio molitor* larvae) during experiments and breeding season monitoring, but do not otherwise receive supplementary feeding or any other management interventions. Testing took place from April to September in 2021 and 2022. Each year, we tested toutouwai in a detour task, an associative learning task, and a reversal learning task. Sample sizes are stated below for each task, with a detailed breakdown provided in electronic supplementary material, table S1.

Experiments were conducted between 9.00 and 15.30, with a consistent testing location set up in a flat, central area of a subject's non-breeding territory. Subjects received up to 20 trials per day, with a freshly killed mealworm used as the reward in all trials. We conducted test sessions on consecutive calendar days, but if this was infeasible (e.g. due to severe weather), testing continued on the next possible day. After completing a trial, birds could begin the next trial as soon as the experimenter had re-baited the apparatus. If a bird did not participate within 2 min in three consecutive trials, testing stopped and was resumed on the next testing day, as this suggested a lack of motivation. An exception to this was if a bird was engaged in important activities such as territory defence. In these cases, the session continued once it returned from the activity.

### 2.1. Detour task

**Figure 1.**
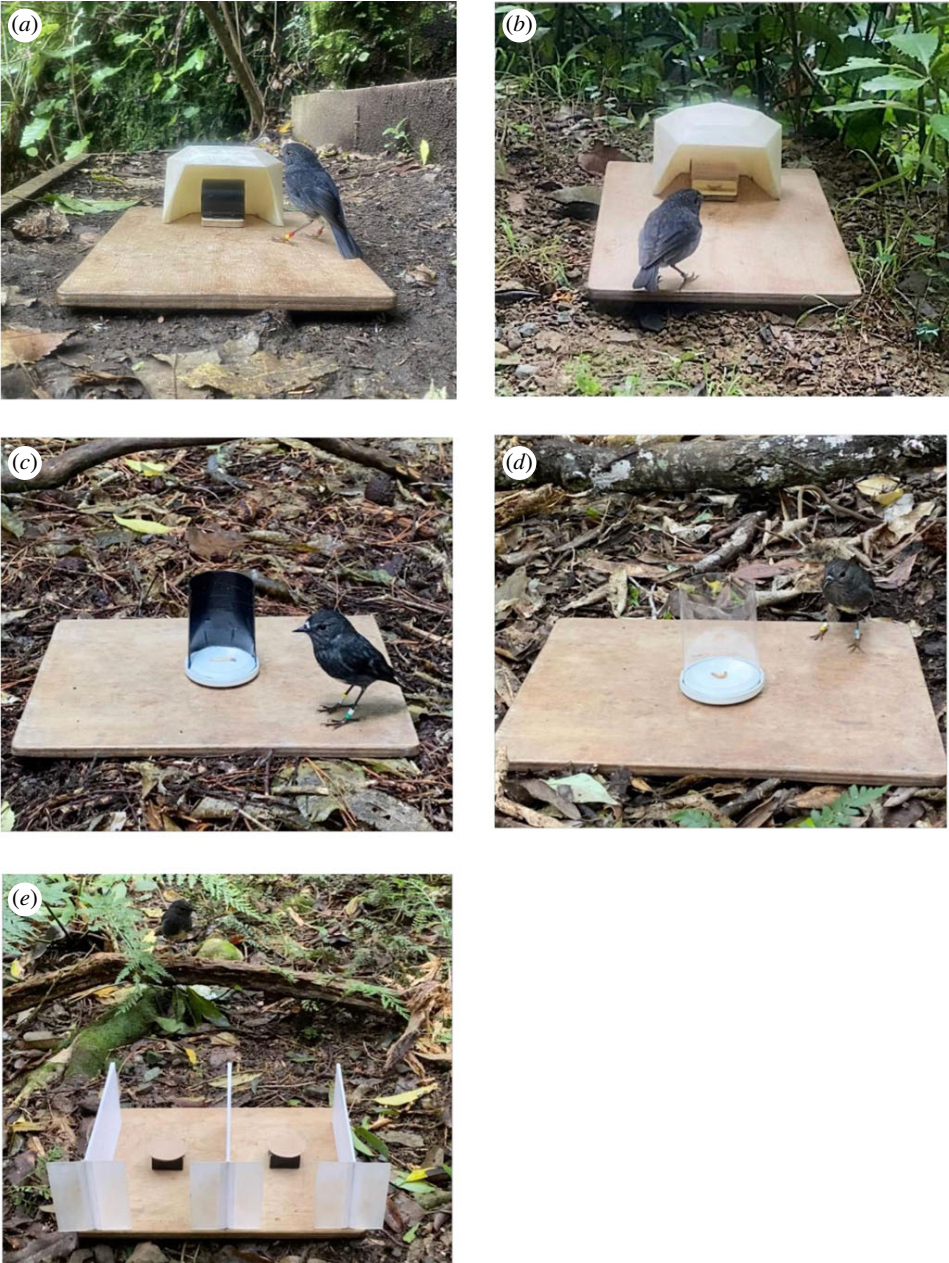
Apparatuses used for cognitive tasks: (*a*) opaque cylinder used for habituation and training in the cylinder detour task, (*b*) transparent cylinder used for the test phase in the cylinder detour task, (*c*) opaque barrier used for habituation and training in the U-barrier detour task, (*d*) transparent barrier used for the test phase in the U-barrier detour, and (*e*) two tiles with removable lids used for the spatial association and reversal learning tasks.

Between April and May 2021, we tested motor inhibition in 45 birds with a cylinder detour task used previously for this species [4]. The apparatus was a plastic cylinder (4 cm diameter × 5 cm length) wrapped in black tape during habituation and training (figure 1*a*), and transparent during testing (figure 1*b*), with a mealworm placed in the centre. A white plastic barrier ensured that subjects first encountered the cylinder face, rather than directly approaching an open end.

We began with a habituation procedure to minimize the effect of neophobia [61]. Here, the opaque cylinder was placed immediately in front of the white plastic barrier so subjects could readily see the mealworm. To pass habituation, a bird needed to take the reward within 2 min in three consecutive trials. Immediately after passing habituation, we began the training phase. During training, the opaque cylinder was placed inside the barrier (figure 1*a*) and the bird learned to reach its head between the barrier wall and cylinder end to retrieve the reward. This ensured that failures during the subsequent test phase were not merely caused by the bird not knowing the detour movement [20]. To pass training, a bird needed to retrieve the mealworm within 2 min in four consecutive trials without first pecking at the cylinder wall.

The test phase began immediately after a bird passed training. The opaque cylinder was replaced with a transparent cylinder (figure 1*b*) so that the mealworm was now visible and subjects could peck directly towards it [20]. To pass the test, a bird needed to inhibit this response and retrieve the mealworm without first pecking at the cylinder in 6/7 consecutive trials (either within or across testing sessions). If a bird did not take the reward within 2 min and did not peck the cylinder, this trial did not contribute to their score, as failure to interact with the apparatus entirely may show that a bird is unmotivated or fearful but does not indicate poor inhibitory control. All individuals who participated in the test phase reached the 6/7 criterion, and their performance score was the number of trials required to achieve this.

From April to September 2022, we tested 45 birds in a second detour task. Thirty-one of these individuals had participated in the cylinder detour task. The apparatus was a plastic U-shaped barrier (9 cm height × 6 cm diameter) mounted on a circular metal base. It was wrapped in black tape during habituation and training (figure 1*c*) and transparent during the test phase (figure 1*d*). A bird needed to detour around the U-barrier to retrieve a mealworm placed in the centre. To prevent the bird from directly approaching the open side of the U-barrier, the experimenter sat facing the open side and only started a trial when the bird was positioned so that they would first encounter the barrier face when moving directly towards the reward. This task invokes the same putative cognitive ability as the cylinder detour, but involves a locomotor detour (moving the whole body around the barrier) rather than the reaching detour (reaching head or limb around the barrier) [20]. We changed the visual appearance and required motor action to reduce the likelihood that prior experience influenced U-barrier task performance, while also allowing us to assess how barrier shape affects detour performance, which has rarely been tested.

During habituation, we placed a single mealworm immediately beside the opaque U-shaped barrier (left/right side order was randomly selected) for the first two trials and behind the barrier in trial three. To pass habituation, a bird needed to retrieve the worm from these locations, each within 2 min. If the bird did not pass a trial, we removed the worm then placed it back in the same location to regain their interest, then began a new trial. Training began immediately after a bird passed habituation, with the mealworm placed inside the opaque U-barrier (figure 1*c*). To pass training, the bird needed to detour around the barrier and retrieve the worm within 2 min in four consecutive trials, without pecking the barrier wall. All birds passed habituation and training.

The test phase began immediately after the bird passed training. The set-up was identical to the training phase except that the barrier was now transparent (figure 1*d*). A bird needed to retrieve a mealworm from behind the barrier without first pecking the barrier wall in 6/7 consecutive trials either within or across test sessions. As with the cylinder task, time-outs without pecking were not counted as failed trials and the performance score was the number of trials a bird required to reach the 6/7 criterion.

### 2.2. Spatial association and reversal learning task

In both 2021 and 2022, we assessed cognitive inhibition using a spatial associative learning task (*N*_2021_ = 41; *N*_2022_ = 37), followed by a spatial reversal learning task (*N*_2021_ = 37; *N*_2022_ = 35). Testing took place between July and September in 2021, and June and August in 2022. Twenty-five birds were tested in both years of the association task, and 21 birds in both reversal learning tasks. In both years, testing began after subjects completed the detour task. For both tasks, the apparatus consisted of two 4 × 4 cm plastic tiles with a 2 cm diameter by 0.8 cm deep well drilled in the centre. These were placed 12 cm apart on a wooden board and separated by three white plastic barriers (21 cm length × 10 cm height). Tiles were covered by brown laminated paper lids (5 cm diameter) to conceal the wells (figure 1*e*).

Prior to testing, we used a behavioural shaping procedure to train subjects to remove lids and retrieve hidden mealworms. In level one, a lid leaned against the tile and the mealworm was visible. In level two, a lid was folded in half so that it formed a tent shape over the tile well and the mealworm was only partially visible in the gap between the tile and lid. In level three, a flat lid covered the tile well and hid the mealworm. To pass each level, a toutouwai needed to take the mealworm in three consecutive trials within 2 min. This necessitated removing the lid in levels two and three. If a bird failed a trial, they regressed to the previous level and needed to pass this again.

For the association learning task, toutouwai learned to associate a spatial location (either the left or right side of the testing apparatus) with a reward. We first gave a bird a spatial preference test and probe trial. In the preference test, the wells of both tiles were baited, and a bird could remove both lids. The location that a bird selected second (i.e. their less preferred location) was then rewarded during their association trials. Next, we gave a bird a probe trial where only one location was baited, and they could remove both lids. As the spatial preference test had rewarded both locations, the probe trial allowed birds to learn that only one location was now rewarded, without this contributing to their overall score. Following the probe trial, they began the association task. Here, only one location was rewarded, and a bird could only remove one lid per trial. Subjects had to choose the rewarded lid on 10/12 consecutive test trials within or across sessions to pass the task (exceeding the chance expectation of 6/12 trials correct, two-tailed binomial test: *p* = 0.039). Birds were given 100 trials to reach this criterion on the association task and 120 trials on the reversal task. The task performance score was the number of trials a bird took to reach the 10/12 criterion. In 2021, two females did not reach criterion after completing 100 trials and so did not move on to the reversal learning task. However, they were given performance scores of 100 and included in analyses of this task, as despite not being an exact score, this indicated that they performed substantially worse than average (see results for the mean number of trials to reach criterion). We also ran all analyses excluding these birds that did not reach criterion, but this did not change the overall results, so we retained them for maximum statistical power. All birds reached criterion in 2022.

The reversal learning task began the day after a bird completed the association learning task. Typically, this was the next calendar day, but this was not always possible. Potentially, individuals with a longer interval between these tasks may have a weaker memory of the association reward location [62], and thus have less difficulty reversing this association. Therefore, to quantify how well birds recalled the rewarded location they had learned in the association task, all birds received three association refresher trials prior to starting the reversal learning task. Here, the mealworm was placed in the same well rewarded in the association learning task. Immediately following this refresher, we began the reversal learning task. For this, the previously rewarded spatial location was now unrewarded and vice versa. Again, a bird was given a probe trial to learn that only one position was rewarded. Subjects then needed to choose the correct location in 10/12 consecutive test trials to pass. The performance score was the number of trials to reach criterion. Four individuals (two per year) did not reach criterion after completing 120 trials. They were given performance scores of 120 and included in analyses. As with the associative learning task, their exclusion did not change the overall results.

#### 2.2.1. Factors affecting task performance

Despite familiarizing subjects with the cognitive tasks during habituation and training, some individuals potentially remained fearful of interacting with the apparatuses and experimenter. Therefore, we tested whether boldness—defined as behavioural responses to risky, but not novel, situations [63]—affected performance during the test phase.

To quantify boldness, we measured subjects' latencies to retrieve a mealworm close to a human experimenter. Neither the food nor experimenter was novel, but the context could still be perceived as risky. To begin a boldness test, the experimenter threw a single mealworm to approximately 1 m in front of their own feet to standardize start distance as much as possible. Immediately after birds ate this mealworm, the experimenter placed a second, freshly killed mealworm 30 cm from their own feet onto a cleared patch of ground. Latency to retrieve this worm was taken as the boldness measure, with a faster latency indicating a bolder individual. In each year, subjects received two boldness tests on consecutive testing days (typically the next calendar day). Twenty-five subjects were tested across both years (2021 and 2022), allowing us to assess whether latency to retrieve the mealworm was temporally repeatable. Significant repeatability would provide evidence that this experiment captured boldness, as behaviour must be consistent across time and context to be considered personality [63]. In 2021, 33 of our subjects received boldness tests in April prior to the detour task, and 11 were tested in July following the detour task. In 2022, subjects (*N* = 37) received boldness tests during July and August following the reversal task. Latencies were coded *in situ* in 2021 and using BORIS software [64] in 2022.

To examine the possibility that latency to retrieve the mealworm during boldness tests may largely measure hunger rather than boldness, birds were given a mealworm on a scale following boldness test sessions in 2022. We divided their mass (measured to the nearest 0.1 g) by their tarsus measurement (mm) to obtain their body condition [4,65]. We also calculated body condition during all cognitive tasks. Again, toutouwai were given a mealworm on a scale after test sessions, and we obtained a single value for body condition in each task by dividing a subject's average mass across all sessions by their tarsus measurement [4,65]. We obtained age data from breeding records. Where subjects were banded as adults (i.e. immigrants or those born before monitoring began in 2014), banding year was used as a proxy for birth year. We determined sex using breeding records, plumage coloration (mature toutouwai are sexually dimorphic [66]), and vocalizations (only males give full-song [67]).

### 2.3. Statistical analysis

We performed statistical analyses in R software [68], using the packages ‘glmmTMB’ [69] to run models, ‘MuMIn’ [70] for model averaging, ‘rptR’ [71] for repeatability estimates, ‘MCMCglmm’ [72] for heritability models, ‘Hmisc’ [73] for correlation matrices and ‘Factoextra’ [74] for principal components analysis (PCA). We follow guidelines from Harper [75] for categorizing the strength of repeatability estimates on a scale from slight (*R* < 0.2) to very high (*R* > 0.9) repeatability.

#### 2.3.1. Relationships between tasks

To explore whether detour and reversal learning tasks demonstrated convergent validity, we used Spearman rank correlation and PCA to compare performances across tasks for individuals who completed all three cognitive tasks during each year (*N*_2021_ = 37, *N*_2022_ = 35). We ran a separate Spearman rank correlation matrix and PCA for task performance in each year, so that only one replicate of each task was included for each bird per year. Only principal components with eigenvalues > 1 were retained in the PCA [76,77]. If individual performances in the detour and reversal task show a significant, positive correlation and load strongly in the same direction onto the same principal component (while the associative learning task loads comparatively weakly or in the opposite direction) this would provide evidence that tasks have convergent validity [2,25].

#### 2.3.2. Factors affecting cognitive task performance

We used linear mixed model-based repeatability analysis to determine whether latency to retrieve a mealworm under threat was temporally repeatable. Latencies were log-transformed to ensure normality. The model included both years of data, resulting in a total of 162 boldness measures across 56 individuals. As individuals were measured twice within each year, we included testing year (2021 or 2022) as a fixed factor to calculate adjusted repeatability. The model also included bird ID as a random factor.

To investigate whether latency to retrieve the mealworm during boldness tests was confounded by hunger, we used a linear mixed model to analyse the effect of body condition on latency to retrieve the mealworm during the 2022 boldness tests (*N* = 51 observations across 32 individuals: not all individuals remained on the scale long enough for a mass measurement). Bird ID was included as a random factor.

We used model averaging to analyse the factors affecting task performance. We expected that non-cognitive factors could have different effects on the U-barrier detour and cylinder detour tasks due to their different apparatuses and required motor actions. For instance, boldness could be more influential for the cylinder task, as birds needed to insert their head in a narrow space between the cylinder and surrounding shield. As our sample size was too small to analyse both detour tasks together and fit interaction terms to account for this, we analysed the factors affecting performance in each detour task (i.e. each year) separately using negative binomial generalized linear models (GLMs) as Poisson models were overdispersed. The association and reversal learning models included both years of task performance as the tasks were identical. We analysed associative learning data and reversal learning data using negative binomial generalized linear mixed models (GLMMs) as Poisson models were overdispersed. We fitted bird ID as a random factor in both GLMMs.

In all models, we included task performance as the response variable, with boldness, body condition, age and sex included as fixed factors. The boldness measure was an individual's average latency to retrieve the mealworm across the two testing days during the year they participated in the task. In the reversal learning task model, we also included the number of refresher trials (out of three) that an individual passed as a proxy for memory of the association reward location. As tarsus measurements, sex or boldness scores were not available for all individuals, we analysed the factors affecting performance for 37 birds in the cylinder detour task, 36 birds in the U barrier detour task, 48 birds for the spatial associative learning task and 46 birds in the spatial reversal learning task.

For the models of the association and reversal learning tasks, we included year as a fixed factor. To test whether prior experience impacted performance, we also ran Mann–Whitney *U*-tests to assess whether experienced birds performed better than naïve birds during the 2022 association and reversal tasks. Similarly, in the U-barrier detour model we included whether an individual was tested in the cylinder task as a fixed factor to assess the effect of prior experience. For each global model described above, we constructed models containing all possible combinations of the predictors. We then averaged parameters across the full model set and calculated 95% CIs and the RVI for each mean *β*. We used model averaging across the full set of possible models of task performance rather than making inferences from a single model, as this can account for model uncertainty and ensure that estimates for fixed effects are robust to different model specifications [78].

#### 2.3.3. Repeatability and heritability of task performance

We assessed the repeatability of detour, association and reversal learning task performance between years by measuring the amount of variation in task performance attributable to differences between-individuals [79]. We ran three separate Poisson GLMM-based repeatability models with task performance as the response variable and bird ID as a random factor. We included repeated measures for birds tested in both years (*N*_detour_ = 62 observations across 31 individuals; *N*_association_ = 50 observations across 25 individuals; *N*_reversal_ = 42 observations across 21 individuals). To increase power, we also included single measures for birds tested in one year only (*N*_detour_ = 28, *N*_association_ = 28, *N*_reversal_ = 30) [80].

We estimated the narrow-sense heritability of task performances using Bayesian GLMM-based animal models. Animal models use a population pedigree to estimate the proportion of additive genetic variance behind a trait. As toutouwai are monogamous [81] and our population has been monitored since 2014 [60], pedigree information was available without genetic analysis. Our pedigree (see the electronic supplementary material) included all subjects tested in our cognitive tasks. We included mother and father ID for test subjects hatched within the study site after monitoring began in 2014. Immigrants and birds hatched before 2014 were included in the pedigree despite unknown parentage, as unlike parent–offspring regression and half-sibling heritability designs, animal models take into account all forms of relatedness between individuals, maximizing statistical power [82]. Animal models can also account for environmental and genetic biases on heritability estimates [82,83].

We ran four models: associative learning task (2021 and 2022), reversal learning task (2021 and 2022), cylinder task and detour task. All models included task performance as the response variable and the following random effects: bird ID linked to the pedigree, bird ID independent of pedigree (to account for permanent environmental effects, which are environmental effects on an individual's phenotype that are constant across repeated measures [83]), mother and father ID (to account for parental effects on offspring phenotype independent of additive genetic effects [84]) and a dominance matrix calculated using the ‘nadiv’ package [85] (to account for genetic dominance effects, which can contribute to non-additive genetic variance [86]). From these models, we estimated *h*^2^ as the additive genetic variance divided by the total phenotypic variance [87]. Models were run using a parameter expanded prior (*V* = 1, nu = 1, alpha.mu = 0, alpha. *V* = 1000) for 500 000 iterations, with a burn in period of 50 000 and a thinning interval of 100 iterations [88].

## 3. Results

### 3.1. Overall performance

The 45 birds that completed the cylinder task in 2021 took between 7 and 24 trials (Mean ± s.d. = 10.0 ± 4.9) to pass habituation and training, and between 6 and 86 trials (Mean ± s.d. = 21.5 ± 20.2) to pass the test. In the 2022 U-barrier detour task, the 45 birds that completed the task took between 7 and 11 trials to pass habituation and training (Mean ± s.d. = 7.1 ± 0.6), and between 6 and 38 trials (Mean ± s.d. = 16.1 ± 8.9) to pass the test.

Birds who reached criterion in the associative learning task required between 10 and 94 trials to pass in 2021 (*N* = 39, Mean ± s.d. = 29.8 ± 19.5) and between 10 and 90 trials in 2022 (*N* = 37, Mean ± s.d. = 26.9 ± 16.5). Birds who reached criterion in the reversal learning task required between 13 and 114 trials in 2021 (*N* = 35, Mean ± s.d. = 50.4 ± 28.9), and between 12 and 94 trials in 2022 (*N* = 33, Mean ± s.d. = 42.1 ± 19.7). Wilcoxon signed rank tests showed that birds who reached criterion in both tasks required significantly more trials to do so during the reversal compared to the association task in both years (2021: *N* = 35, *Z* = −3.645, *p* < 0.001; 2022: *N* = 33, *Z* = - 3.664, *p* < 0.001).

### 3.2. Relationships between tasks

There were no significant correlations between performances on the detour, association or reversal learning task in 2021 or 2022 (table 1). One component with an eigenvalue > 1 was extracted from the PCA of task performances in both years, explaining 49.8% of the variation in task performance in 2021 and 47.4% in 2022. In both years, detour task performance loaded moderately and positively onto PC1, while association and reversal learning task performance loaded moderately and negatively (table 2).

**Table 1.** Results of Spearman correlation for task performances in 2021 (*N* = 37) and 2022 (*N* = 35).

			reversal learning task	detour task
2021	detour task	*r*_s_	−0.064
		*p*	0.708
	associative learning task	*r*_s_	0.253	−0.102
		*p*	0.130	0.547
2022	detour task	*r*_s_	−0.295
		*p*	0.085
	associative learning task	*r*_s_	0.216	−0.049
		*p*	0.214	0.782

**Table 2.** Results of principal components analysis for task performances in 2021 (*N* = 37) and 2022 (*N* = 35).

task	2021	2022
	PC1	PC1
detour task	0.363	0.527
associative learning task	−0.669	−0.550
reversal learning task	−0.649	−0.648
eigenvalue	1.494	1.423
% variance explained	49.796	47.432

### 3.3. Factors affecting task performance

During boldness tests, toutouwai showed low but significant repeatability in their latency to retrieve the mealworm whilst adjusting for testing year (*R* ± s.e. = 0.281 ± 0.084, 95% CI = 0.121–0.443, *p* < 0.001). Body condition had no effect on latency to retrieve the mealworm during the 2022 boldness tests (*β* ± s.e. = 2.031 ± 4.474, 95% CI = −6.715 to 10.865, *p* = 0.653).

We found no effect of boldness, or any other non-cognitive factor we examined (e.g. age, sex, body condition), on performance in the detour (electronic supplementary material, tables S2 and S3), association (electronic supplementary material, table S4) or reversal learning tasks (electronic supplementary material, table S5). Moreover, prior experience did not improve performance: birds who participated in 2021 did not outperform naive birds during the associative learning task in 2022 (*Z* = −0.032, *p* = 0.974), reversal learning task (*Z* = −1.008, *p* = 0.313) or U-barrier detour task (electronic supplementary material, table s3). Support for the top three models of performance in each task is shown in electronic supplementary material, table S6.

### 3.4. Repeatability and heritability of task performance

**Figure 2.**
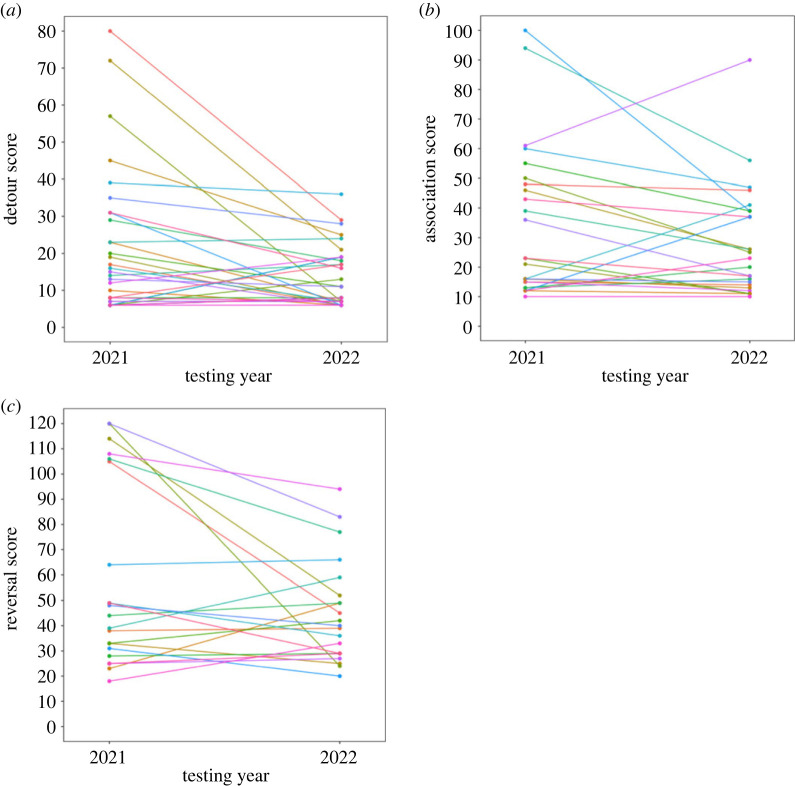
Relationship between individual performances in 2021 and 2022 on the (*a*) detour task, (*b*) associative learning task, and (*c*) reversal learning task. Circles represent individual performances with repeated measures connected by lines.

Toutouwai showed low to moderate repeatability in their detour performance between years and barrier shape (*R* ± s.e. = 0.360 ± 0.153, 95% CI = 0.042–0.676, *p* = 0.024) (figure 2*a*). They also showed moderate to high between-year repeatability in their performance in the association task (*R* ± s.e. = 0.649 ± 0.133, 95% CI = 0.342–0.804, *p* < 0.001) (figure 2*b*) and moderate repeatability in the reversal learning task (*R* ± s.e. = 0.511, 95% CI = 0.147–0.738, *p* = 0.009) (figure 2*c*). However, there was no evidence for heritability of performance in the cylinder detour (*H*^2^ = 0.051, 95% CI = 0.000–0.186), U-barrier detour (*H*^2^ = 0.084, 95% CI = 0.000–0.272), association (*H*^2^ = 0.097, 95% CI = 0.000–0.360) and reversal learning tasks (*H*^2^ = 0.105, 95% CI = 0.000–0.307). 95% credible intervals indicated substantial uncertainty surrounding all estimates, and lower bounds did not differ from zero [88].

## 4. Discussion

We found no association between an individual's performance on detour and spatial reversal learning tasks. However, performance within each of these tasks was robust to potential confounds and repeatable across years. This suggests that detour and reversal learning tasks are reliable cognitive measures but are not underpinned by the same ability. Yet despite individual consistency in task performance, we found no evidence for heritability, potentially suggesting that inhibitory control is a plastic trait in toutouwai.

### 4.1. Relationships between tasks

We found no significant correlations between task performance measures, yet our PCA results suggested that the association and reversal learning tasks were at least partly underpinned by the same ability. This is expected, since both required individuals to form a spatial association using the same apparatus. By contrast, PCA results indicated that detour and reversal learning tasks captured distinct abilities. Both tasks evoked the expected prepotent responses and therefore seemed to require inhibitory control. Toutouwai readily learned the detour motor action when the barrier was opaque, but regularly pecked at the transparent barrier during the test phase [20]. Likewise, they required more trials to pass the reversal phase compared with the association, suggesting that they struggled to inhibit acting on their knowledge of the previously rewarded location [7]. However, despite these superficial similarities, the lack of correlation between task performances suggests that the suppression of prepotent physical responses (motor inhibition) is a distinct cognitive ability from the suppression of prepotent mental representations (cognitive inhibition) in toutouwai. Alternatively, as the reversal learning task required cognitive flexibility and spatial associative learning in addition to inhibitory control [22,89], any shared reliance on inhibitory control during the detour and reversal tasks may have been too small to detect during our study. Additionally, as tasks were conducted at different times of year, it is possible that temporal behavioural variation affected performance and obscured correlations between tasks. However, toutouwai do not show any major behavioural differences across the non-breeding season [59,90], which is when testing took place. Unmeasured confounding variables, such as other aspects of personality, may have also affected each task differently and masked positive relationships between them [25]. However, the literature overwhelmingly supports a lack of convergent validity across a range of inhibitory control tasks in non-human animal species, including dogs [2,26,28], birds [29,91], primates [25] and lizards [27]. Therefore, it seems likely that our data suggest that inhibitory control is not a unitary construct in toutouwai, with motor and cognitive inhibition functioning as separable components of this ability.

### 4.2. Factors affecting task performance

An ongoing concern in inhibitory control research is the extent to which tasks capture non-cognitive variables [3,4]. Reassuringly, we found no effect of boldness, body condition or prior experience on any task performance. Likewise, we did not find an effect of sex or age. Two previous studies have assessed the influence of body condition on cylinder detour performance in toutouwai, using very similar tasks but producing conflicting results. Shaw *et al.* [60] found no correlation between condition and performance, but a later study with a larger sample size found that birds in better condition required fewer trials to pass [4]. Interestingly, the average number of trials toutouwai required to reach criterion in [4] was roughly half that of Shaw *et al.* [60] and the current study. This suggests that subtle differences in the environment or experimental procedures may have affected how toutouwai performed in [4], leading to better performance and generating a body condition effect. These inconsistent findings highlight a more general problem with replicability in the cognitive sciences [92], one that is particularly relevant to field experiments where standardization is exceedingly difficult [93].

Birds who participated in the cylinder detour task did not perform significantly better than naive birds on the U-barrier detour the following year, a finding corroborated by previous studies of toutouwai [4] and guppies [94]. While prior experience improved performance on a novel detour task three days later in pheasants [3], toutouwai do not appear to remember the properties of transparent barriers after 1 year, or are unable to generalize their knowledge to a visually distinct apparatus requiring a different motor action [20]. Similarly, although animals can improve their performance over multiple reversals conducted in quick succession [95,96] potentially by adopting a win-stay/lose-shift strategy and anticipating upcoming reversals [22], experience on the association/reversal learning tasks did not improve task performance after a 1-year interval in toutouwai.

Toutouwai showed low but significant repeatability in their latencies to take a mealworm under threat. Although we did not measure contextual repeatability and thus cannot be sure that we captured a personality trait, this temporal consistency does provide some evidence that our test measured boldness [63]. Furthermore, body condition did not affect latency to retrieve the mealworm, suggesting that this test was not simply measuring hunger. Despite boldness being a predictor of task performance in other species [32–34], we found no evidence for this in toutouwai. We anticipated that shyer individuals may be more fearful during testing, which can impair inhibitory control [36]. However, our habituation procedures may have ensured that the apparatus and experimenter were no longer perceived as risky during testing [17]. Equally, there was little indication that personality led to selection bias in experiments. In each year, we tested the majority of toutouwai in the study population, and in only one case was a bird unwilling (i.e. potentially too shy/neophobic) to interact with the apparatus.

### 4.3. Repeatability and heritability of task performance

In all tasks, toutouwai were repeatable in their performance across a 1-year interval. Consistency was highest for the associative learning task (*R* = 0.649), followed by the reversal learning task (*R* = 0.511) and the detour task (*R* = 0.360). All values exceeded the average temporal repeatability of cognitive performance found in a meta-analysis by Cauchoix *et al.* [38] (*R* = 0.18–0.28). The lower repeatability observed in the detour task may be because the tasks were visually distinct and required different locomotor actions. Equally, our results may indicate that associative learning ability (or more specifically, spatial associative learning) is more temporally stable than inhibitory control, supported by the fact that reversal learning (putatively capturing both associative learning and inhibition) was less repeatable than associative learning alone. Similar repeatability results were found in wild Australian magpies, where repeatability over a 3-year period was strongest for associative learning, somewhat lower for reversal learning, and non-significant for a detour task [41]. However, it should also be noted that repeatability values for task performance may not necessarily reflect the true repeatability of cognitive traits [38]. We cannot exclude the possibility that our estimates also captured consistency (or lack thereof) in non-cognitive confounds of tasks performance that were unmeasured in our study [97]. Nonetheless, our results do provide some evidence that these tasks captured consistent individual differences in cognition.

Surprisingly, Shaw [4] found no repeatability over a 1-year interval for cylinder detour tasks in toutouwai. However, the smaller sample size used in this previous study (15 individuals tested twice, compared to 31 in the current study) may have reduced the reliability of parameter estimates [79]. Toutouwai in this previous study also performed substantially better with less inter-individual variation during their second task presentation compared to the current study. This may have affected repeatability estimates, as these measure the amount of inter-individual variation relative to intra-individual variation [39].

Despite finding consistent individual variation in all tasks, we found no evidence that task performance was heritable. Heritability estimates were low and non-significant for all tasks, ranging from 0.051 to 0.105. However, the precision of these estimates was poor: 95% credible intervals indicated that true heritabilities could range from negligible to moderate [88]. This may be a consequence of our small sample size and missing pedigree information for some subjects, both of which affect the power to detect heritability using animal models [98]. Our results are in line with studies of Aegean wall lizards (*Podarcis erhardii*) and great tits, which found no evidence for heritability in reversal learning task performance [49,50]. By contrast, reversal learning performance showed moderate heritability in red junglefowl (*Gallus gallus*) [51] and house mice (*Mus musculus*) [53], as did detour task performance in pheasants [52]. Since repeatability was high compared with heritability, inhibitory control variation may be largely shaped by environmental factors in toutouwai, the effects of which remained relatively stable over the 1 year period that repeatability was assessed [39,43].

Plasticity of inhibitory control performance in non-human animals has been reported by other studies. Aegean wall lizards from less variable physical environments were better at reversal learning [99] but this trait shows no heritability in this species [49]. Conversely, pheasants reared in spatially unpredictable environments showed better detour performance [100]. Social factors can also shape inhibitory control development. Group size was positively related to detouring ability in spotted hyaenas (*Crocuta crocuta*) [101], and Australian magpies from larger groups performed better on a suite of cognitive tasks, including detour and reversal learning [5]. Toutouwai are largely solitary [59], so social factors may be comparatively unimportant for this species. However, characteristics of the physical environment could explain the stable cognitive differences we observed, as toutouwai often retain the same territory for life [102].

Obtaining more robust heritability estimates and exploring non-genetic influences on inhibitory control will be a valuable avenue for future research in toutouwai. Additionally, exploring relationships between inhibitory control and fitness would be helpful in interpreting our heritability estimates, since traits important to fitness often show low heritability due to strong directional selection depleting genetic variation [39,103]. Toutouwai of both sexes cache to manage short-term fluctuations in food availability [104], and males share food with their mates during courtship and incubation [59]. Both behaviours likely require birds to inhibit eating food immediately, so inhibitory control may predict survival and reproductive success in this species when selection favours caching and food-sharing over immediately eating food as a behavioural tactic. However, our low heritability estimates for all tasks (albeit preliminary) suggest that the cognitive traits underpinning performance would evolve relatively slowly in toutouwai, even if they were subject to strong selection [43].

## 5. Conclusion

Despite increasing interest in the evolution of inhibitory control, little is known about the long-term repeatability and heritability of this ability in the wild or the validity of widespread testing paradigms. In wild toutouwai, we found no evidence for a ‘general’ inhibitory control ability underpinning performance across detour and reversal learning tasks, supporting the growing consensus that inhibitory control is best conceptualized as an umbrella term capturing distinct abilities in many species. Multi-task approaches will be necessary to understand the evolution of inhibitory control in species where this is not a unitary construct, since we cannot assume that all components have the same genetic basis or relationship to fitness. Despite this, detour and reversal learning tasks appear to be valuable measures of motor and cognitive inhibition, respectively, in toutouwai, as performance was repeatable across years and unaffected by potential confounds. However, we found no evidence that task performance was heritable, suggesting that individual differences in inhibitory control may be plastic in this species. Our study highlights the importance of dissecting what cognitive tasks measure, as this determines the conclusions we can draw about the causes and consequences of cognitive variation.

## Data Availability

Supplementary material is available online [105].
